# The application of strong matrix management and PDCA cycle in the management of severe COVID-19 patients

**DOI:** 10.1186/s13054-020-02871-0

**Published:** 2020-04-17

**Authors:** Yuanchao Li, Hongliang Wang, Jundong Jiao

**Affiliations:** 1 Department of Critical Care Medicine, The Second Affiliated Hospital of Harbin Medical University, Harbin, Heilongjiang China; 2 Heilongjiang Province Medical Aid Group for CVOID-19, Wuhan, Hubei China; 3 Department of Nephrology, The Second Affiliated Hospital of Harbin Medical University, No. 246, Xuefu Road, Nangang District, Harbin, 150001 Heilongjiang China

In late December 2019, an outbreak of 2019 novel coronavirus (COVID-19) was reported in Wuhan, Hubei province [1], with a rapid transmission [2]. As of 24:00 March 19, there were still 6569 confirmed cases including 2136 severe patients. A total of 80,967 confirmed cases and 3248 deaths were reported [3]. The intensive medical staffs from Heilongjiang province came to Wuhan Union Hospital, Tongji Medical College of Huazhong University of Science and Technology**,** to support. We did well with this emergency strong matrix management (SMM) mode and Plan-Do-Check-Act (PDCA) cycle in the management of severe COVID-19 patients for more than 50 days. Therefore, we summarized the application of this emergency management mode. We hope it can be helpful in dealing with the outbreak of COVID-19 and managing severe patients.

## The problem we faced

The patients we treated were all severe and critical [4]. We faced difficulties and challenges that we had never met before.

1. Cooperate with the local medical team and adapt to unfamiliar working environment and workflow.
2. Most of the patients were middle-aged and elderly patients [5] with distinct local accents. Some of them could not take care of themselves. Communication barriers made our work more difficult.
3. The understanding about COVID-19 was limited. The treatment protocol for severe patients was uncertain [6].
4. How to ensure the timely and effective transmission of patient information in isolation?
5. How to arrange working intensity and working time reasonably to reduce the infection risk of medical staffs?
6. The mental stress of the medical staffs increased significantly.

## Coping strategies

### Application of the SMM mode to operate efficiently

**Fig. 1:**
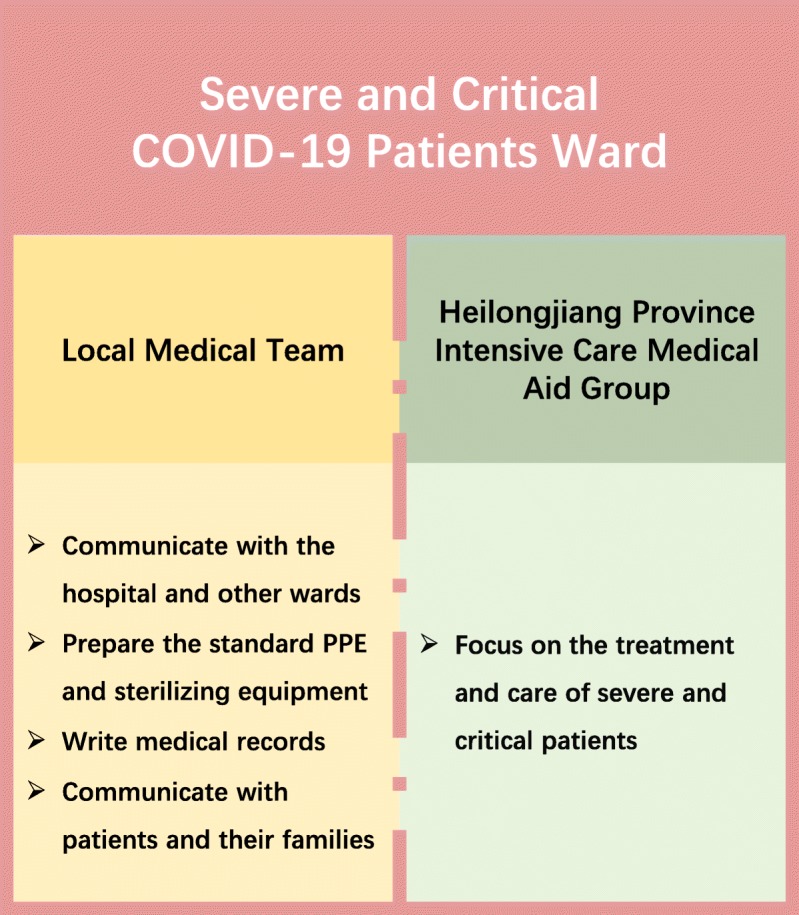
The SMM mode. SMM, strong matrix management; COVID-19, coronavirus disease 2019; PPE, personal protective equipment

In order to ensure efficient operation in the shortest time, we decided to break the original division of hospital functions and adopt the SMM model (Fig. 1). SMM refers to an organizational framework established with both vertical organizational hierarchies and horizontal relationships across departmental lines that pool team members together for specific work assignments or projects. A matrix management plan directs team members to the assignment where the need and the benefit derived are the greatest. Based on the SMM mode, the medical staffs from Union Hospital were mainly responsible for the communication with the hospital and other wards, the preparation of standard personal protective equipment (PPE) and sterilizing equipment, the writing of medical records, and the communication with patients and their families to release mental stress. The intensive medical staffs focused on the treatment and care of severe patients. Both teams gave full play to their respective advantages and carried out their duties to fight against the epidemic together.

### Application of the PDCA cycle to improve medical quality

**Fig. 2:**
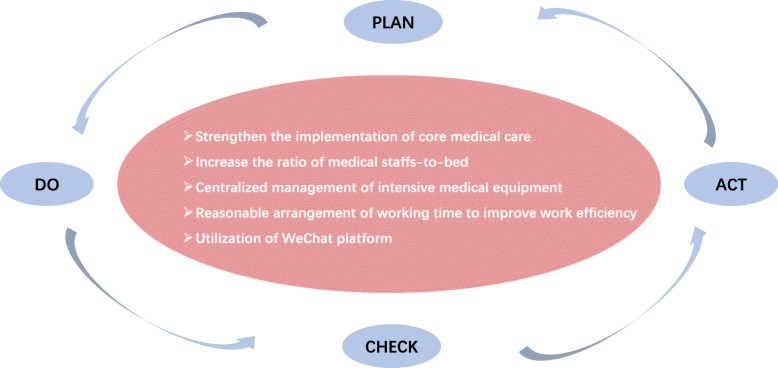
The application of PDCA cycle. PDCA, Plan-Do-Check-Act

We applied the PDCA cycle in the anti-epidemic emergency management process in intensive care areas (Fig. 2). We followed plan-execution-inspection-modulate cycle repeatedly to summarize, analyze, modify, and re-execute constantly. The stepped cycle mode forwards the medical quality in the management of severe patients.

#### Strengthen the implementation of core medical care

The medical round system required that the chief physician made at least four grand rounds a week and the associate chief physician (team leader) made at least two rounds a day.

Medical studies were organized weekly. Death cases were discussed within 24 h. The treatment group discussed the condition of critical patients daily. Difficult cases were timely discussed in the ward with all the doctors in order to find out the problems and solve in time.

Consultation system was actively implemented, including consultation from other wards or experts from other medical teams to cope with patients’ comorbidities and complications. Give full play to the advantages of multidisciplinary treatment (MDT).

#### Increase the ratio of medical staffs-to-bed

Studies have already confirmed that the number of available medical staffs and beds in the intensive care unit (ICU) will significantly affect the mortality of critical patients [7, 8]. So we adjusted the ratio of medical staffs-to-bed. For critical patients, we increased the doctor-to-bed ratio to 1.5–2:1 and the nurse-to-bed ratio to 4:1. For severe patients, the doctor-to-bed ratio was 0.5–0.7:1 and the nurse-to-bed ratio was 0.8–1:1.

#### Centralized management of intensive medical equipment

The isolation ward was temporarily transformed according to the routine requirements of “three areas and two roads” for infectious diseases. There were a total of 50 beds in the ward, among which 6 beds were set for critical patients. There were 8 invasive respirators, 6 noninvasive respirators, 6 high flow nasal cannula instruments, 1 blood gas analyzer, and 1 blood purification machine in the intensive care area.

#### Reasonable arrangement of working time to improve work efficiency

The number of medical staffs should be limited in emergency situations on the premise of ensuring medical quality. We arranged a flexible shift system, with nurses on duty every 4 h and doctors on duty every 6 h. According to the work intensity, the medical staffs of Union Hospital and our intensive medical team worked together to complete each on-duty shift.

#### Utilization of WeChat platform

In order to ensure timely and effective communication between the isolation ward and the outside, we also utilized effective WeChat platform in addition to fixed telephone and walkie-talkie. The patients’ vital signs and changes in the conditions and treatment were promptly sent to the medical working group by means of text, pictures, and videos.

### Targeted psychological counseling

We actively launched training to help team members get familiar with the local working environment and workflow. Strict infection control training and supervision of the implementation reduced the mental stress of team members who are concerned about being infected by COVID-19 [9].

## Conclusion and perspective

Medical treatment system is a core part for the prevention and control of public health emergencies. The intensive medical staffs from Heilongjiang province established a new emergency management strategy by applying the SMM mode and PDCA cycle to fight against the epidemic of COVID-19 in Hubei province. “There are a thousand Hamlets in a thousand people’s eyes”. Each medical team adopted a corresponding protocol to deal with COVID-19 according to their own situation. We hope to help achieve a comprehensive epidemic prevention victory worldwide.

## Data Availability

All data generated or analyzed during this study are included in this published article.

## References

[1] Huang C, Wang Y, Li X, Ren L, Zhao J, Hu Y (2020). Clinical features of patients infected with 2019 novel coronavirus in Wuhan, China. Lancet.

[2] Wang D, Hu B, Hu C, Zhu F, Liu X, Zhang J, et al. Clinical characteristics of 138 hospitalized patients with 2019 novel coronavirus-infected pneumonia in Wuhan, China. JAMA. 2020. 10.1001/jama.2020.1585. 10.1001/jama.2020.1585 PMC7042881 32031570

[3] Coronavirus COVID-19 Chinese Cases by National Health Commission of the PRC. http://fms.news.cn/swf/2020_sjxw/2_1_xgyq/index.html?v=0.8438956046482258. Accessed 20 Mar 2020.

[4] National Health Commission. Diagnosis and treatment plan of COVID-19 (trial 7th edition). 2020.http://www.nhc.gov.cn/yzygj/s7653p/202003/46c9294a7dfe4cef80dc7f5912eb1989/files/ce3e6945832a438eaae415350a8ce964.pdf. Accessed 20 Mar 2020.

[5] Wang W, Tang J, Wei F (2020). Updated understanding of the outbreak of 2019 novel coronavirus (2019-nCoV) in Wuhan, China. J Med Virol.

[6] Wang M, Cao R, Zhang L, Yang X, Liu J, Xu M (2020). Remdesivir and chloroquine effectively inhibit the recently emerged novel coronavirus (2019-nCoV) in vitro. Cell Res.

[7] Wunsch H, Angus DC, Harrison DA, Collange O, Fowler R, Hoste EA (2008). Variation in critical care services across North America and Western Europe. Crit Care Med.

[8] Landrigan CP, Rothschild JM, Cronin JW, Kaushal R, Burdick E, Katz JT (2004). Effect of reducing interns’ work hours on serious medical errors in intensive care units. N Engl J Med.

[9] Phan LT, Nguyen TV, Luong QC, Nguyen TV, Nguyen HT, Le HQ (2020). Importation and human-to-human transmission of a novel coronavirus in Vietnam. N Engl J Med.

